# Rapid growth of retroperitoneal adipose leiomyoma after inguinal hernia mesh repair: An observation case report suggesting a potential association with mesh material

**DOI:** 10.1097/MD.0000000000043594

**Published:** 2025-07-25

**Authors:** Ming-Hao Jia, Wen-Xu Liu, Chuan-Ying Li, Jia-You Xu

**Affiliations:** a School of Clinical Medicine, Shandong Second Medical University, Weifang, Shandong, China; b Department of Gastrointestinal Surgery, The First Affiliated Hospital of Shandong Second Medical University, Weifang, Shandong, China.

**Keywords:** complication, inguinal hernia, lipoleiomyoma, mesh material, retroperitoneal

## Abstract

**Rationale:**

Lipoleiomyomas are rare benign tumors typically characterized by slow growth. However, this case demonstrates an unusually rapid enlargement of a retroperitoneal lipoleiomyoma following hernia mesh repair, highlighting a potential link between synthetic mesh materials and tumorigenesis.

**Patient concerns:**

A 47-year-old female presented with a rapidly enlarging right lower abdominal mass detected 1 month after undergoing laparoscopic right inguinal hernia mesh repair. The tumor grew from an initial size of 5 cm to 15 cm × 10 cm within 6 months, raising concerns about malignancy or mesh-related complications.

**Diagnoses:**

Pelvic enhanced CT revealed a large mass (15 cm × 10 cm) in the right lower anterior abdominal wall and pelvis. Postoperative pathological examination confirmed the diagnosis of a benign lipoleiomyoma.

**Interventions:**

Surgical excision was performed, with the tumor found at the site of prior mesh implantation.

**Outcomes:**

Complete resection was achieved, and the patient recovered without complications. Histopathology confirmed a lipoleiomyoma with no malignant features.

**Lessons:**

This case suggests that lipoleiomyomas may exhibit rapid growth in rare instances, warranting close postoperative surveillance. The tumor’s location at the mesh implantation site raises the possibility of mesh-induced chronic inflammation contributing to tumorigenesis, necessitating further investigation into the long-term effects of synthetic meshes.

## 1. Introduction

Lipoleiomyoma is a rare benign tumor and a rare variant of leiomyoma. It is composed of a mixture of smooth muscle cells and adipocytes, with an incidence of 0.03% to 0.2%.^[1]^ Although these tumors are usually slow growing and have a good prognosis, they can occasionally show an abnormally rapid growth rate, causing significant clinical symptoms and usually requiring surgical treatment. We report the case of a 47-year-old woman who underwent laparoscopic mesh repair of a right inguinal hernia and rapidly developed a large retroperitoneal lipoleiomyoma in the area of mesh implantation. Based on a detailed analysis of this case and a literature review, this paper puts forward a new consideration: the potential association between lipoleiomyoma and mesh materials after inguinal hernia repair, which provides a new perspective and perspective for clinical practice.

## 2. Case presentation

**Figure 1.**
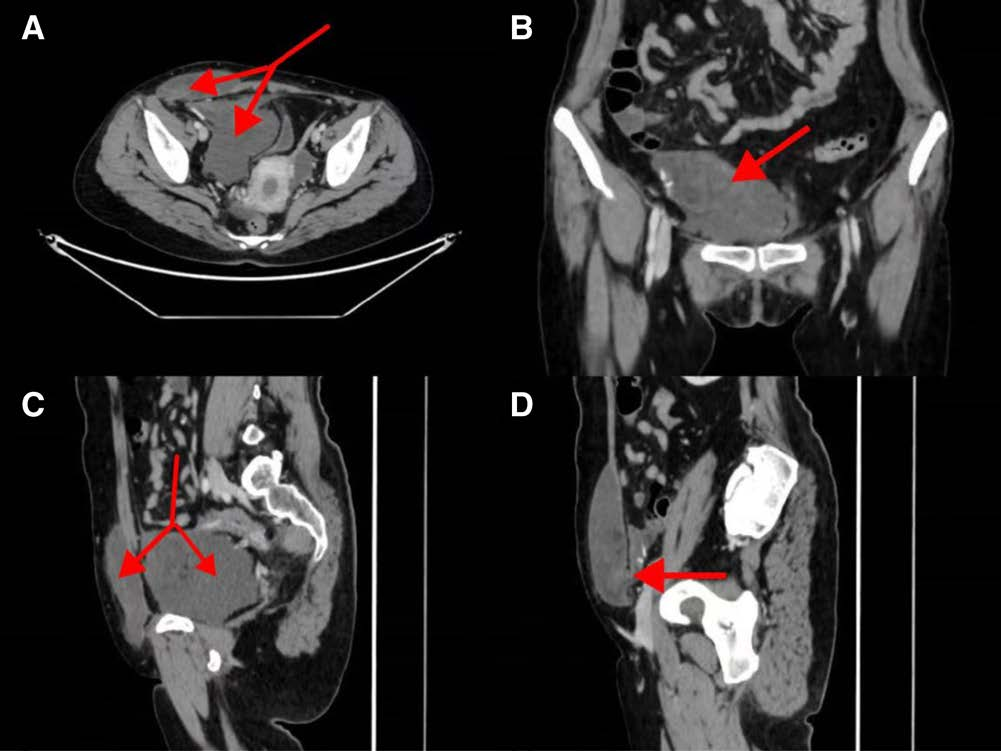
Abdominal computed tomography (CT) showed an irregular mass (arrows) from the right lower abdominal wall to the right side of the pelvis, with uneven density, fat density shadows, and strip vascular shadows. The volume was approximately 15 cm × 12 cm.

**Figure 2.**
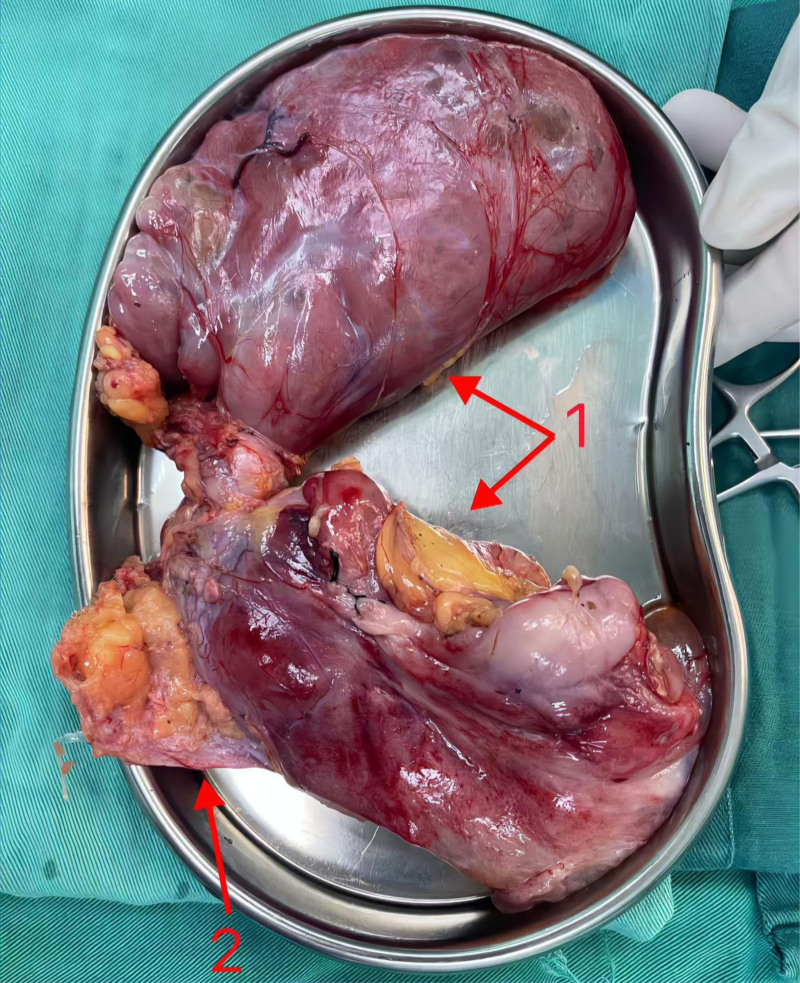
Tumor and mesh of the case. Arrow 1: tumor; Arrow 2: mesh. Note: image resolution was limited by original intraoperative; key structures are labeled to maximize clarity.

**Figure 3.**
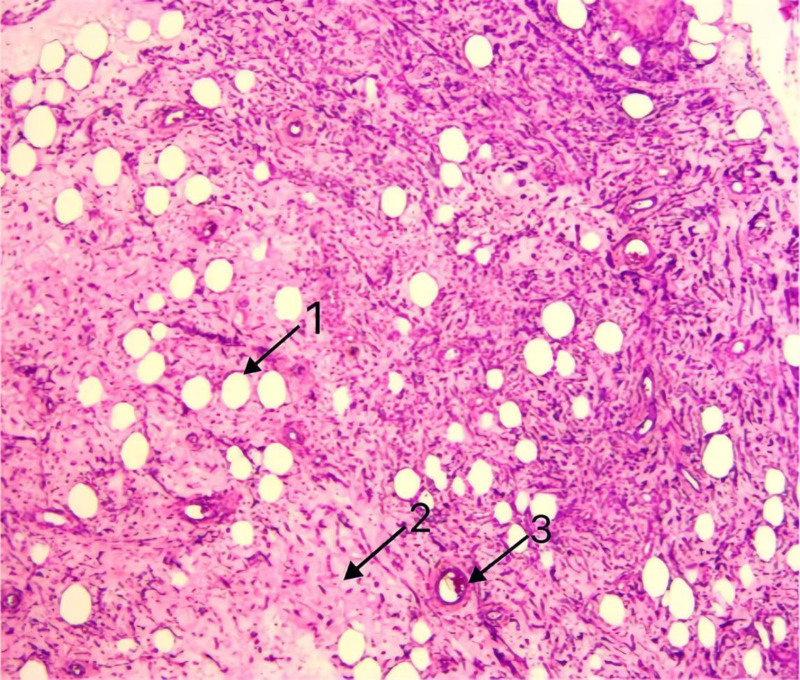
Pathological findings of the case. Arrow 1: large number of mature adipocytes; Arrow 2: smooth muscle cells arranged in fascicles interlaced 169. Note: image resolution was limited by pathological records; key structures are labeled to maximize clarity.

A 47-year-old female patient underwent laparoscopic right indirect inguinal hernia mesh repair (TAPP) using polypropylene mesh material in June 2023 (The specific brand of polypropylene mesh used during the initial hernioplasty could not be identified as the procedure was performed at an external institution). During the operation, the abdominal cavity was routinely explored, and no abnormal masses were found. One month after the surgery, the patient inadvertently touched a mass in the right lower abdomen without any accompanying symptoms and did not care. Subsequently, the mass gradually increased in size and pain developed in the right lower quadrant. On March 30, 2024, the patient was admitted to the Department of Gastrointestinal Surgery, Weifang People’s Hospital. Enhanced CT of the abdomen and pelvic CT showed an irregular mass from the right lower abdominal wall to the right side of the pelvis, with uneven density and a volume of approximately 15 × 12 cm (Fig. 1). Laparoscopic resection of the abdominal lesion was performed on April 02, 2024. Intraoperative exploration showed that the retroperitoneal mesh could be seen in the right inguinal region, and the Retzius, internal ring, and Bogros spaces were obviously raised, partially cystic, predominantly solid, encapsulated mass. Endoscopic dissection was difficult and the patient was converted to open surgery. An oblique incision (approximately 15 cm long) was made in the right inguinal region, and the skin of the incision was explored. The subcutaneous cystic mass was approximately 15 × 10 cm, soft in texture, containing viscous fluid and visible material of unknown nature, extending down from the inner ring of the groin. The mass was carefully separated along its edge, and the aponeurosis of the external oblique muscle and transverse fascia of the abdomen were dissected in layers. The tumor gradually separated along its edge. The tumor was located in the retroperitoneal area, extending upward along the Bogros space, up to the level of the anterior superior iliac spine, close to the external iliac bloodvessels and hip bone on the outside, tightly attached to the mesh and peritoneum on the inside, and extending inward and downward along the Retzius space, inside the bladder and below the pubic symphysis, with a size of approximately 15 × 10 cm. The tumor was carefully separated along the edge of the tumor, the adhesion mesh and peritoneum were removed, and the tumor was completely dissected (Fig. 2). Postoperative pathology showed amesenchymal tumor (18 × 11 × 2.5 cm) with abundant bloodvessels and surrounding local foreign body granuloma formation (Fig. 3). Immunohistochemical results showed that ER (+), PR (+), SMA (+), desmin (+), calretinin (−), WT (+) – 1, CD34 (−), S-100 (+) a few, CK (−), CD117 (in 71+), HMB45 (−), MelanA (−), and P16 (−), Ki-67 (+3%). The patient recovered well after the surgery and was discharged on April 10, 2024. No mass was found on abdominal ultrasonography 6 months later.

## 3. Discussion

The most common site of lipoleiomyoma is the uterine body, while other rare sites include the cervix, ovary, broad ligament, and retroperitoneal space. It is more common in perimenopausal and postmenopausal women and is often accompanied by metabolic disorder-related diseases, such as obesity, diabetes, and hypothyroidism.^[2]^ Akbulut et al reported 70 patients with lipoleiomyoma with a mean age of 55.49 years and most of whom were postmenopausal.^[3]^ In this case, the patient was 47 years old at the time of diagnosis, and the patient had regular menstruation before menopause, and was in good health with a BMI of 20.94 kg/m2(The patient’s height upon entering the hospital was 160 cm and the weight was 53.6 kg), no significant weight gain in half a year, and no metabolic abnormalities. This finding is inconsistent with the above reports and indicates the presence of incidental cases. This case suggests that lipoleiomyoma may occur in patients without typical risk factors (such as metabolic disorders or menopausal status), and the differential diagnosis should be expanded during clinical diagnosis.

At present, the pathogenesis of lipoleiomyomas remains unclear, and the most widely recognized theory is cell metaplasia. Uterine muscle cells have the potential to differentiate into adipocytes. In addition, undifferentiated mesenchymal stem cells in the myometrium can differentiate into adipocytes to form a lipoleiomyoma.^[4]^ Estrogen deficiency and metabolic disturbances may also play a role in inducing and promoting the proliferation of lipoma components in lipoleiomyomas.^[5]^ The decrease in estrogen secretion in perimenopausal and menopausal women may promote the proliferation of adipocytes in lipoleiomyomas, leading to progressive enlargement of uterine lipoleiomyoma after menopause. The patient in this case had no significant medical history and laboratory data showed no metabolic disorders.

Patients with lipoleiomyomas are usually asymptomatic in the early stages, and the lesions are usually incidentally touched or found on imaging studies. The clinical symptoms are related to the size and location of the tumor, and there maybe abdominal pain, abnormal vaginal bleeding, and other symptoms. If the tumor is large, the mass can be palpable in the lower abdomen, and compression symptoms of the bladder and rectum, such as dysuria and constipation, can occur.^[6]^ Although lipoleiomyomas are benign tumors, no morphological features can predict the outcome and growth rate of lipoleiomyomas. The rapid growth of this patient’s tumor within 7 months is unusual. Therefore, regular review of tumor size and assessment of growth stability are necessary to adjust the treatment plan.

Imaging is very important for the diagnosis of lipoleiomyomas. Ultrasound, computed tomography (CT), and magnetic resonance imaging (MRI) are the main diagnostic tools. They help to determine the location, size, and scope of the lesion and provide a reference for the development of treatment plans. Ultrasound can quickly detect the size and location of tumors and has the characteristics of safety, no radiation, low price, and is often used as the first choice. CT can show the situation around the tumor, but the resolution of the soft tissue is low. MRI has a high resolution of soft tissue and can evaluate the extent of tumor invasion and the relationship between adjacent structures, which is helpful for a smooth operation. However, it cannot be distinguished from lipomas or liposarcoma.^[6]^ Pathological examination is the gold standard for diagnosing lipoleiomyomas. Under a microscope, fat cells or tissues were mixed with leiomyoma cells. On immunohistochemistry, lipoleiomyomas were positive for smooth muscle actin, desmin, and vimentin, but negative for HMB45 and CD34.^[7]^ Mignogna et al also reported the immunoreactivity of adipocytes to vimentin, desmin, and SMA, which supports the hypothesis that smooth muscle cells are directly converted into adipocytes.^[8]^

**Figure 4.**
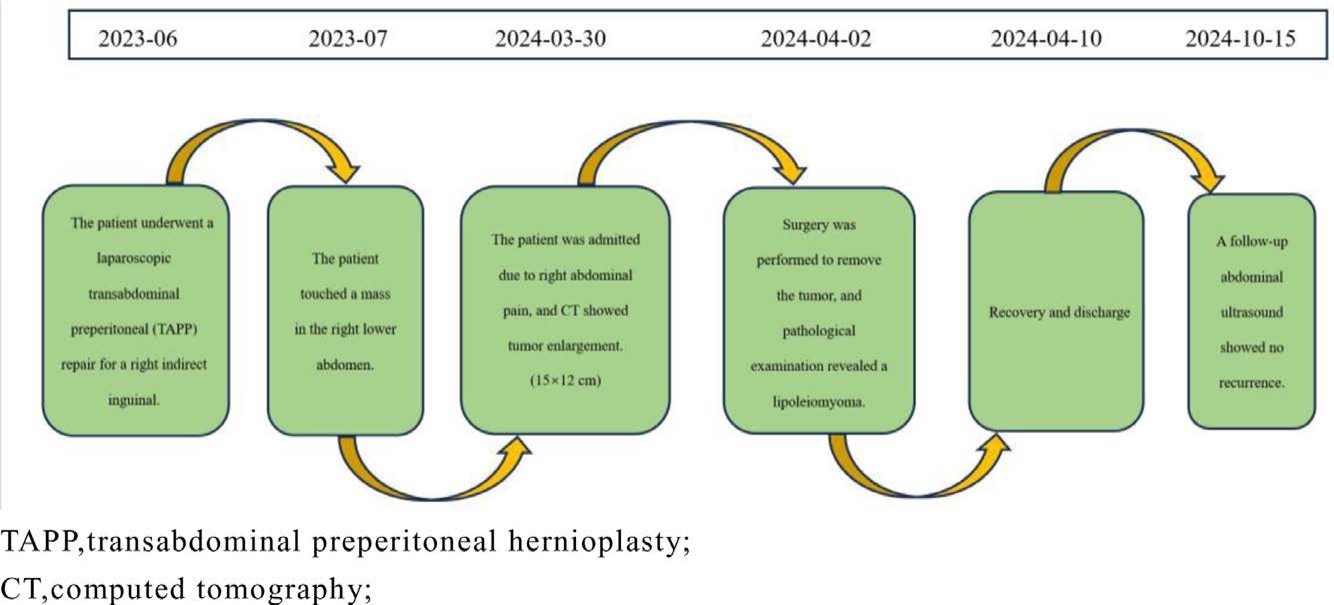
The timeline of relevant diagnoses and treatments. TAPP = transabdominal preperitoneal hernioplasty, CT = computed tomography.

Typically, lipoleiomyomas that are asymptomatic and clinically similar to leiomyomas do not require specific treatment; however, a follow-up review is warranted to check for changes in tumor size or symptoms. If the tumor grows rapidly, the abdominal organs are affected or compressed, and the corresponding clinical manifestations appear, complete surgical resection should be performed. The prognosis of lipoleiomyomas is excellent and recurrence or metastasis is rare. However, McDonald et al reported 3 cases of uterine liposarcoma related to lipoleiomyoma of the uterus, suggesting that the possibility of malignant transformation of uterine lipoleiomyoma also requires regular follow-up.^[9]^ The timeline of relevant diagnoses and treatments is showcased in Figure 4.

Although there is no definite research report to support a direct correlation between mesh materials and lipoleiomyoma, in this case, the tumor occurred in the area of mesh implantation for inguinal hernia surgery, so we cannot completely exclude the possibility of a correlation between the mesh and tumorigenesis through a continuous inflammatory response or other potential mechanisms after implantation of the mesh as a foreign body material.

It has been shown that the implantation of foreign body mesh materials in vivo can induce a series of biological reactions, such as inflammatory cell infiltration, fibrosis, angiogenesis and so on.^[10]^ These responses could theoretically lead to local cellular gene mutations or oncogene activation, which in turn could induce tumorigenesis. Artificial mesh implantation can cause persistent foreign body reactions and inflammation, and a large number of bone marrow-derived immune cells infiltrate the implanted area.

Heymann et al showed that polypropylene mesh implantation results in rapid and sustained monocyte/macrophage infiltration with complement activation, cytokine release, and matrix remodeling for at least 90 days in a mouse hernia repair model.^[11]^ Although most patients have no obvious clinical symptoms, a long-term inflammatory microenvironment may promote tumorigenesis.

Therefore, it cannot be completely excluded that the occurrence of lipoleiomyoma in this case was related to the persistent foreign body reaction and inflammation caused by mesh implantation during the previous inguinal hernia surgery. Owing to the low incidence of lipoleiomyomas, more studies are needed to elucidate the potential association between mesh material implantation and tumorigenesis. If such an association exists, it will have important theoretical significance and clinical guidance value in reducing the incidence of complications after hernia repair.

## 4. Conclusion

Although lipoleiomyomas are benign tumors, abnormal growth behavior may still occur in individual cases, and close follow-up is needed. It is worth further discussion that the tumor in this case occurred in the area of mesh implantation for inguinal hernia surgery, and we speculate that mesh material implantation may be involved in the occurrence of the tumor through mechanisms such as persistent foreign body reactions and inflammatory responses. Therefore, we recommend further studies on the relationship between the mesh material and tumorigenesis to reduce postoperative complications.

## Author contributions

**Data curation:** Ming-Hao Jia.

**Formal analysis:** Chuan-Ying Li.

**Investigation:** Ming-Hao Jia, Wen-Xu Liu, Chuan-Ying Li.

**Resources:** Ming-Hao Jia.

**Supervision:** Jia-You Xu.

**Validation:** Jia-You Xu.

**Writing – original draft:** Ming-Hao Jia.

**Writing – review & editing:** Ming-Hao Jia, Jia-You Xu.
